# Computational and Experimental Characterization of dVHL Establish a Drosophila Model of VHL Syndrome

**DOI:** 10.1371/journal.pone.0109864

**Published:** 2014-10-13

**Authors:** Merav D. Shmueli, Lee Schnaider, Gal Herzog, Ehud Gazit, Daniel Segal

**Affiliations:** Department of Molecular Microbiology and Biotechnology, George S. Wise Faculty of Life Sciences, Tel Aviv University, Tel Aviv, Israel; Weizmann Institute of Science, Israel

## Abstract

The von Hippel-Lindau (VHL) cancer syndrome is associated with mutations in the *VHL* gene. The pVHL protein is involved in response to changes in oxygen availability as part of an E3-ligase that targets the Hypoxia-Inducible Factor for degradation. pVHL has a molten globule configuration with marginal thermodynamic stability. The cancer-associated mutations further destabilize it. The *Drosophila* homolog, dVHL, has relatively low sequence similarity to pVHL, and is also involved in regulating HIF1-α. Using *in silico*, *in vitro* and *in vivo* approaches we demonstrate high similarity between the structure and function of dVHL and pVHL. These proteins have a similar fold, secondary and tertiary structures, as well as thermodynamic stability. Key functional residues in dVHL are evolutionary conserved. This structural homology underlies functional similarity of both proteins, evident by their ability to bind their reciprocal partner proteins, and by the observation that transgenic pVHL can fully maintain normal dVHL-HIF1-α downstream pathways in flies. This novel transgenic *Drosophila* model is thus useful for studying the VHL syndrome, and for testing drug candidates to treat it.

## Introduction

The von Hippel-Lindau (VHL) syndrome is a rare hereditary cancer, associated with mutations in the *VHL* tumor suppressor gene. It is characterized by increased susceptibility to various tumors, both benign and malignant, including central nervous system haemangioblastomas, renal cysts and renal cell carcinoma (RCC) and phaeochromocytoma [1]–[3]. The pVHL tumor suppressor protein is the substrate recognition subunit of a complex, comprising pVHL as well as Elongin C and B, termed VCB complex [4]–[6]. This complex functions as part of an SCF-like ubiquitin-ligase that promotes the degradation of target proteins required for growth and vascularization of solid tumors [7]–[9]. The pVHL protein has been implicated in a variety of cellular processes, most notably in response to changes in oxygen availability, due to its role as part of an E3-ligase which targets the Hypoxia-Inducible Factor (HIF1-α) for proteasomal degradation [10]–[12]. Upon decrease in oxygen levels residues P564 and/or P402, in the oxygen dependent degradation domain (ODD, residues 401–603) of HIF1-α, are hydroxylated and interact with Y98 in pVHL leading to VCB-mediated E3 degradation of HIF1-α [13]–[15]. This hydroxylation reaction is mediated by the prolyl-hydroxylase domain protein (PHD; [16]). Under hypoxic conditions, the prolyl hydroxylase is inactive and the HIF1-α subunit is not hydroxylated [17]–[19]. HIF1-α then dimerizes with the β subunit (HIF1-β), which is constitutively expressed. The HIF heterodimer translocates to the nucleus where it functions as a transcription factor [20]. The best known target genes of HIF encode proteins involved in glycolysis, glucose transport (Glut-1), angiogenesis (vascular endothelial growth factor (VEGF)) and erythropoiesis (erythropoietin), i.e., proteins that mediate the cellular response and adaptation to hypoxic conditions [21]–[24]. The *VHL* gene encodes two biologically active isoforms of pVHL (19 kDa and 30 kDa) as a result of in-frame alternative AUG codon usage [25]–[27]. However, no functional significance has been assigned to the extra 53 amino acids at the N-terminus of the large isoform. pVHL shuttles back and forth between the nucleus and the cytoplasm [28], [29]. Some pVHL is also found in mitochondria and is also associated with the endoplasmic reticulum [30], [31]. The short isoform of pVHL is localized primarily to the nucleus whereas the long isoform is more frequently associated tightly with microtubules in the cytoplasm [32], [33].

The first crystal structure of pVHL, bound to the VCB complex, was determined in 1999 and shed a light on its domains and structure [25]. Other studies of pVHL, unbound to its partners (Elongin B and C), revealed a molten globule conformation which was found by biophysical analyses to have marginal stability [34]. Recently, pVHL was characterized as an intrinsically disordered protein (IDP; [35]). It has been proposed that the IDP nature of various proteins facilities their interaction with a variety of proteins and complexes by allowing multiple conformations [36], [37]. This feature may aid pVHL in binding its various partners and in carrying out its multiple cellular functions [38].

VHL is evolutionarily conserved from worms to mammals [39]. The *Drosophila* VHL (dVHL) was identified by amino-acid sequence homology and lacks the N-terminal extension found in the 30 kDa isoform of the human pVHL [40]. The dVHL protein was shown to interact with the *Drosophila* Elongin C homolog and could also form complex with the human VCB complex [41]. The domain corresponding to ODD in the fly homolog of HIF1-α (termed SIMA) spans its amino acid residues 692–863 and contains a conserved proline residue (P850) which has been proposed to mediate the oxygen-dependent stabilization of SIMA as do P564 and/or P402 in the human HIF1-α [42], [43]. Notably, dVHL was shown by pull down assay to bind a GST-tagged ODD peptide of either the human HIF1-α or its *Drosophila* homolog, SIMA, under normoxic conditions, providing further evidence for its functional evolutionary conservation [43].

In order to gain structural insight into the apparent conservation of the function of dVHL and pVHL we generated *in silico* a model structure for dVHL which revealed overall structural similarity between the two proteins and more significantly, evolutionary conservation of the key functional amino acids in pVHL and dVHL and their orientation within the secondary structures of the proteins.

We corroborated these observations by *in vitro* biophysical assays under physiological conditions which showed that pVHL and dVHL are similar in their secondary and tertiary structures, thermodynamic stability and molten globule conformation. This structural homology between pVHL and dVHL may underline their reported functional homology in binding the VCB complex [41]. Indeed we determined that they similarly bind *in vitro* both the human and *Drosophila* derived ODD peptide. We further examined this homology *in vivo* in the context of the intact organism using transgenic *Drosophila*. We found that pVHL can rescue, via the HIF dependent pathway, the mutant phenotype of loss of function of dVHL.

Taken together our results indicate that the transgenic *Drosophila* model generated accurately represents the Mammalian pVHL-HIF1-α pathway and is thus a useful tool for studying the VHL syndrome and VHL function in a whole organism.

## Results

### Comparison of the human and *Drosophila* VHL proteins

**Figure 1:**
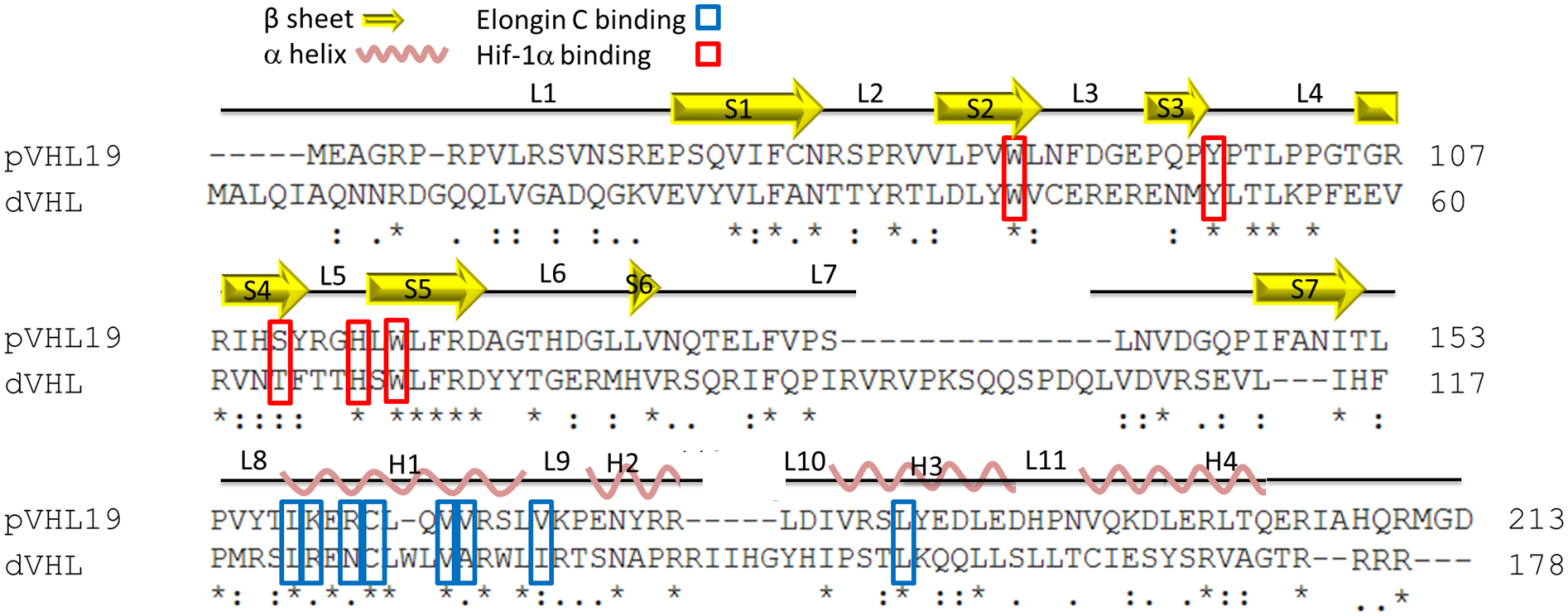
Sequence alignment of pVHL and dVHL, the alignment was generated using the MUSCLE algorithm, and shows structural motifs (β strands and α helices) of pVHL and conservation of the Cul2 and BC boxes as well as residues involved in Hif-1α binding. *(asterisk) - fully conserved residue: (colon) - similar residue, scoring >0.5;. (period) weakly similar residue, scoring = <0.5.

Human pVHL and *Drosophila* dVHL have been shown to have similar functions through their E3 ubiquitin ligase activity [41]. To assess the structural basis for the functional similarity between the two proteins, we searched for structural similarities between the human and fly homologues. Although the structure of human pVHL has already been elucidated by crystallographic methods, this has yet to be achieved for *Drosophila* dVHL. We therefore built an *in silico* three-dimensional model for *Drosophila* dVHL. In addition to their similar functions, pVHL and dVHL belong to the same superfamily according to the Pfam database, suggesting that they are evolutionarily related and may share the same folding [44]. Indeed, two fold-recognition algorithms, FFAS03 and HHpred, identified pVHL19 as a structural template for dVHL [45], [46]. The relatively low sequence identity between pVHL19 and dVHL (<22% sequence identity and <30% similarity as calculated using the MUSCLE algorithm [47] Figure 1) required the use of a composite modeling approach in order to optimize the alignment between the target (dVHL) and template (pVHL19) sequences. Our modeling procedure employed fold recognition algorithms such as FFAS03 and HHpred with additional sequence alignments computed by the MUSCLE algorithm. We integrated hydrophobicity and evolutionary conservation algorithms and the final model was produced using MODELLER [48]–[53]. Structural data regarding the pivotal amino acids for pVHL19 interactions with its binding partners were used for alignment and orientation of amino acids within the model. Additional validation was achieved by comparison of our model structure to that produced by standard modeling tools such as I- TASSER [54] and Phyre2 [55]. The model structure produced by our composite approach shares significant structural similarity to these additional models (Figures S1 and S2).

**Figure 2:**
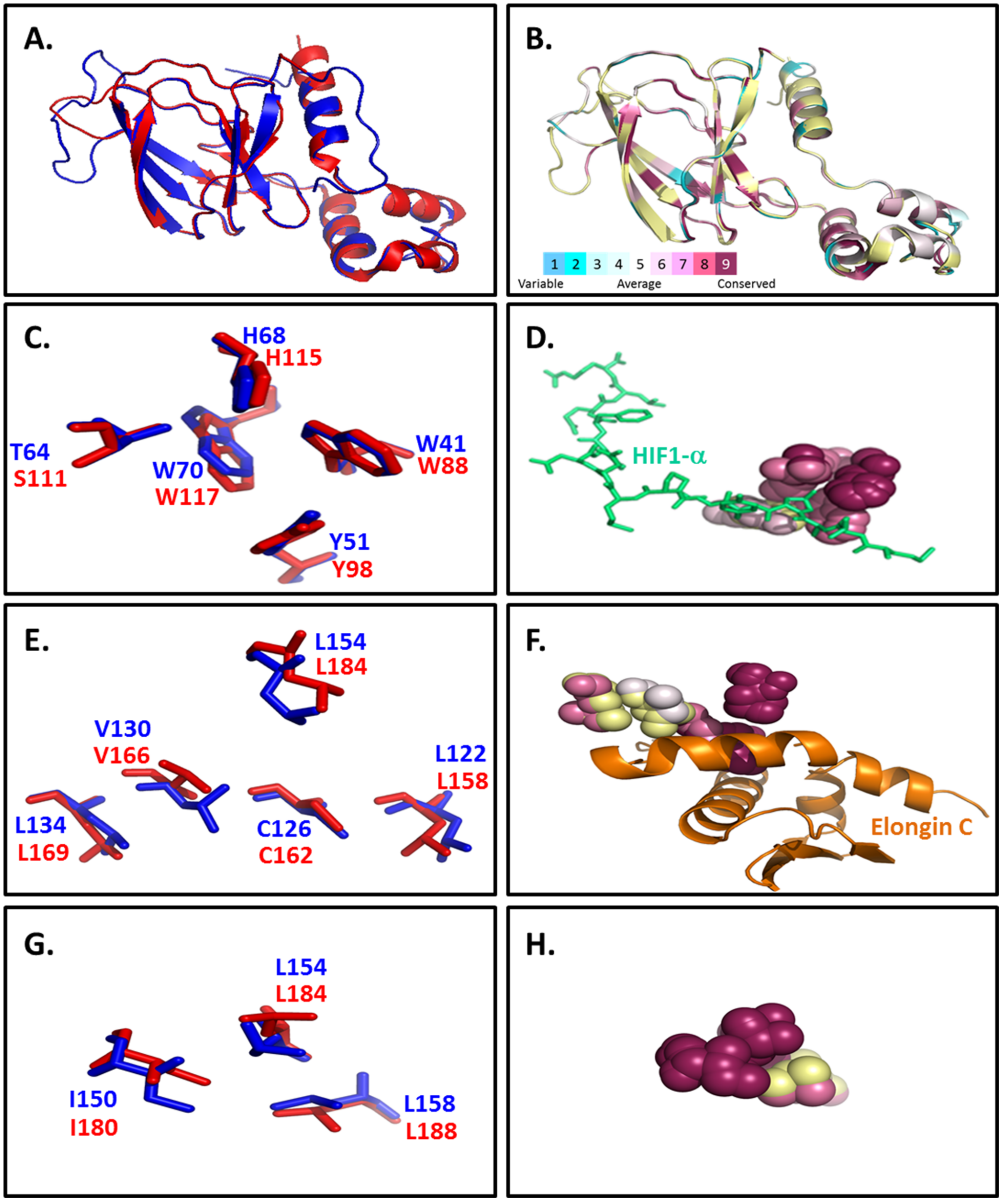
Comparison of the orientation and evolutionary conservation of the key functional residues of pVHL and dVHL. (**A**) Superposition of the structure of pVHL (red) (PDB ID: 1lm8) and the dVHL model structure (blue). (**B**) Superposition of the structure of pVHL (PDB ID: 1lm8) and the dVHL model structure, showing evolutionary conservation scores as calculated by ConSurf. (**C**) Superposition of the key amino acids involved in HIF1-α binding in pVHL (red) and dVHL (blue) (these are residues W117, H115, S111, Y98 and W88 in pVHL and W70, H68, T64, Y51and W41 in dVHL). (**D**) Superposition of the key amino acids involved in HIF-1α binding in pVHL and dVHL, showing evolutionary conservation of these residues, as calculated by ConSurf. HIF1-α shown in green. (**E**) Superposition of the key amino acids involved in Elongin C binding in pVHL (red) and dVHL(blue) (these are residues L184, L169, V166, C162 and L158 in pVHL and L154, L134, V130, C126 and L122 in dVHL). (**F**) Superposition of the key amino acids involved in Elongin C binding in pVHL and dVHL, showing evolutionary conservation of these residues, as calculated by ConSurf. Elongin C shown in orange. (**G**) Superposition of the key amino acids involved in Cul2 binding in pVHL (red) and dVHL (blue) (these are residues L188, L184 and I180 in pVHL and L158, L154 and I150 in dVHL). (**H**) Superposition of the key amino acids involved in Cul2 binding in pVHL and dVHL, showing evolutionary conservation of these residues, as calculated by ConSurf.

Superimposition of the resultant dVHL model structure on the crystal structure of pVHL19 (PDB ID: 1LM8) shows that the overall domain structure of pVHL19 is conserved in dVHL (Figure 2A). The β domain structure, which consists in pVHL of a seven-stranded β sandwich (residues 1–116) and an α helix (residues 154 to 165), is overall more conserved than the α domain in the dVHL model structure. The main difference between the β domain of the two structures is the absence of the smallest of the seven β strands of pVHL (S6) in the dVHL model structure, which is therefore predicted to consist of a six-stranded β sandwich and an α helix. The α domain in pVHL is comprised of three α helices (residues 122 to 159). One of these three helices in dVHL is predicted to assume a random coil conformation as the amino acid sequence which makes up this region in dVHL does not favor helix formation (Figure 2A).

The comparison between the two structures also provided hints for the functional conservation of pVHL and dVHL with regard to the binding of HIF1-α and the interactions with Elongin B, Elongin C and Cul2 (Figure 2B). HIF1-α is known to interact exclusively with the β domain of pVHL by binding alongside its β sandwich [25]. The formation of the complex between HIF1-α and pVHL is mediated primarily by the interactions between a hydroxyproline (Hyp564) of HIF1-α and the following amino acids in pVHL: W88, Y98, W117, H115 and S111. These amino acids are not only conserved in the linear sequence of dVHL (S111 in pVHL is replaced by T64 in dVHL) but are part of the same secondary structures and assume the same orientation in the dVHL model as they are in the pVHL crystal structure (Figure 2C). The orientation of these amino acids is crucial for the function of pVHL and predictably of dVHL as well. Furthermore, we found that Y98 and W117 (Y51 and W70 in dVHL) are predicated to be highly evolutionary conserved, in agreement with the fact that mutations of these residues abolish HIF1-α binding (Figure 2D).

**Figure 3:**
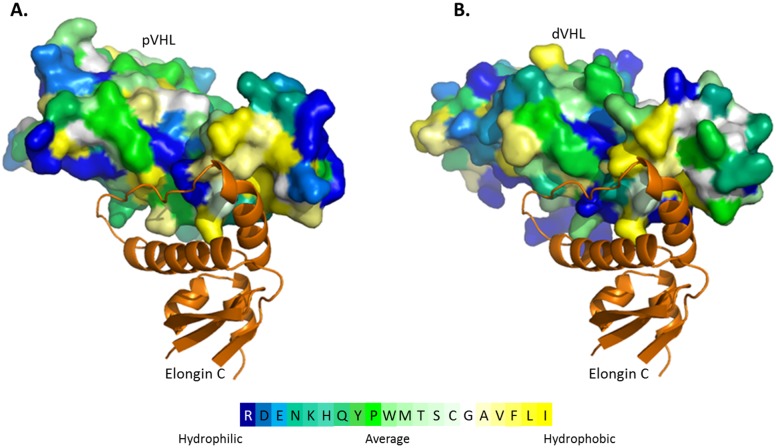
Comparison of the hydrophobicity of pVHL and dVHL. The interface between pVHL (surface) and Elongin C (cartoon, orange) is greatly hydrophobic, with only a few key hydrophilic residues in the periphery. This pattern is conserved in dVHL. (**A**) Hydrophobicity of pVHL (PDB ID: 1LM8) shown with its binding partner human Elongin C. (**B**) Hydrophobicity of the dVHL model structure superimposed with human Elongin C (from PDB ID: 1LM8).

The pVHL-Elongin C interface is almost completely hydrophobic with only a few significant hydrogen bonds at the periphery [25]. pVHL and dVHL share similar hydrophobicity on a whole and in their α-domains specifically, which comprise of the amino acids shown to be important for Elongin C binding (Figure 3). Interestingly, sequence alignment between human Elongin C and *Drosophila* Elongin C shows that hydrophobicity between the two is highly conserved as well (Figure S3). The H1 helix of the α-domain of pVHL makes extensive contacts with Elongin C. The most significant van der Waals contacts are made by L158, C162 and R161 and these are augmented by contacts from the K159, V165, V166 and L169 side chains of pVHL. The other two helices of the α-domain of pVHL also contribute contacts to Elongin C, with L184 making the most extensive ones in this region. The majority of these amino acids are conserved not only in the linear sequence of dVHL but are also oriented in dVHL similarly to their orientation in pVHL, even though the amino acids of H2 in dVHL are predicted to assume a random coil structure (Figure 2E). In addition, these corresponding dVHL residues are also evolutionary conserved (Figure 2F).

Finally, pVHL has been shown to interact with Cul2. It is proposed that this is achieved via six amino acids that make up the Cul2 box in the pVHL sequence [56]. Out of these six residues, four are conserved in dVHL and three adopt very similar orientation and are evolutionary conserved (Figure 2G, H).

To conclude the modelling analysis, despite relatively low sequence conservation between human pVHL and fly dVHL, these two proteins are predicted to adopt the same fold. Moreover, the pivotal functional amino acids in pVHL and dVHL are oriented in very similar positions, allowing the formation of the intricate network of interactions which constitute the interfaces of the VCB complex. Taking these results into consideration with the fact that the key functional amino acids are also evolutionary conserved in pVHL and dVHL, we postulate that the two orthologous proteins may be able to interact with each other’s binding partners.

We examined the similarity in folding and stability of the two proteins *in vitro* as well as the ability of the two proteins to regulate each other’s downstream HIF dependent pathways *in vivo*.

### Folding and stability of dVHL are similar to pVHL

Several biophysical methods were employed to compare folding and stability of human pVHL and its *Drosophila* homolog dVHL.

**Figure 4:**
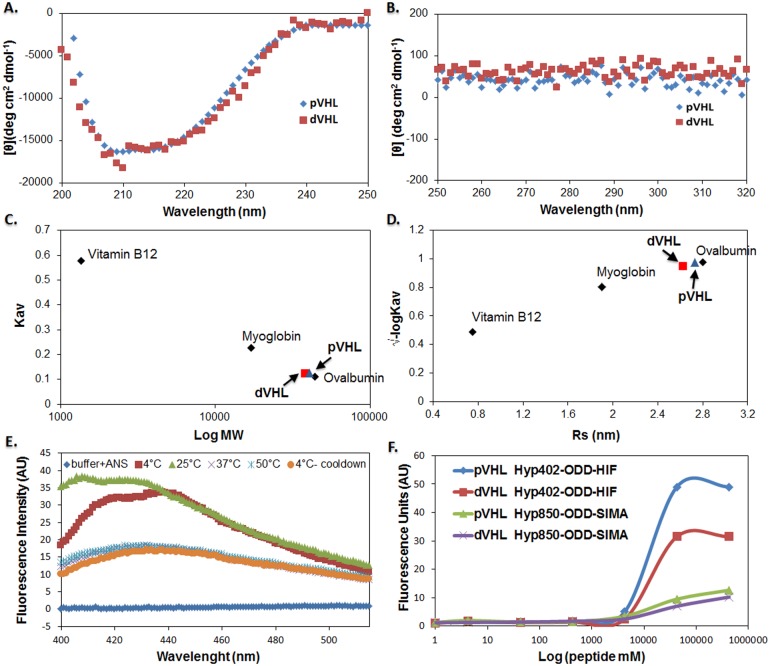
Folding, stability and function of VHL monitoring by biophysical methods. (**A, B**) **Circular dichroism:** Far-UV (A) and near-UV (B) CD spectra of pVHL (blue) and dVHL (red). CD spectra measurements were conducted at 25°C. Protein sample was at final concentration of 5 µM in 10 mM Tris-HCl (pH 8) and 200 mM NaCl. (**C, D**) **Size exclusion chromatography:** preformed at 10 mM Tris-HCl (Ph 8) and 200 mM NaCl. (C) Calibration curves generated for molecular weight standards (Kav versus Log MW). Apparent molecular mass of pVHL (blue) and dVHL (red) was calculated with the curve equation (C). Apparent R_S_ of pVHL (blue) and dVHL (red) were calculated with the curve equation (√-logKav versus R_S_) (D). Molecular weight standard proteins (black): Vitamin B12 (1.35 kDa, 0.75 nm), Myoglobin (17 kDa, 1.9 nm), Ovalbomin (44 kDa, 2.8 nm), γ-globulin (158 kDa, 5.1 nm) and Thyroglobulin (670 kDa, 8.6 nm). (**E**) **ANS fluorescence studies:** Comparison of spectrum measured with ANS alone (blue), and spectrum obtained following addition of 3 µM dVHL at different temperatures (4, 25, 37 and 50°C) and after cool down (E). (**F**) **Functional analysis of dVHL and pVHL:** Binding of dVHL or pVHL to TAMRA labeled Hyp402-ODD-HIF or Hyp850-ODD-SIMA target peptides (F). Fluorescence of TAMRA at 580 nm was measured as a function of log of peptide concentration (mM). The results are presented after subtraction of the negative control (BSA with peptide at different concentrations). pVHL or dVHL protein concentration was 500 nM; pVHL incubated with Hyp402-ODD-HIF peptide (blue), pVHL incubated with Hyp850-ODD-SIMA peptide (red), dVHL incubated with Hyp402-ODD-HIF peptide (green) and dVHL incubated with Hyp850-ODD-SIMA peptide (purple).

We examined the conformational motifs, including α-helices, β-sheets and turns by Circular Dichroism (CD) spectra of soluble, bacterially expressed pVHL and dVHL, unbound to their Elongin B, C and HIF1-α partners by far UV (178–250 nm) at 25°C. This analysis indicated that pVHL and dVHL have similar secondary structure comprising of both α-helices and β-sheets, estimated by the K2D2 algorithm to represent 32% and 34% of pVHL, respectively and 14% and 32% of dVHL, respectively (Figure 4A; Table 1). These values for pVHL are in agreement with those previously reported for this protein [34].

**Table 1:** Analysis from k2d2 program of pVHL and dVHL suggest similar secondary structure content of α-helix, β-sheet and random structure.

	pVHL	dVHL
**α-helix (%)**	14	29
**β-sheet (%)**	32	34
**Random (%)**	54	37

We next evaluated the tertiary structures of the two proteins by near-UV CD spectra (205–350 nm) at 25°C. No significant signals originating from aromatic side chains were observed for either protein, suggesting a dramatic loss of the tertiary structure of the soluble and unbound dVHL and pVHL (Figure 4B). This data indicates that both proteins are in molten globule state since the signal is near zero.

The thermodynamic stability of dVHL was assessed via thermal denaturation measurements by CD. The melting temperature (*T_m_*) of dVHL was found to be about 45°C (Figure S4A), identical to the *T_m_* reported for pVHL [34]. Furthermore, the unfolding of dVHL was only partially reversible (Figure S4B), and formation of amorphous aggregates could be visualized by the naked eye, as reported for pVHL [34].

Size exclusion chromatography (SEC) was next used to estimate the hydrodynamic dimensions and the compactness of the tertiary structure of pVHL and dVHL. Elution volume of partially or fully unfolded proteins is significantly smaller than that of well folded proteins due to the large increase in the Stokes radius (R_s_). The dVHL protein eluted at a retention volume of 10.38 ml and pVHL at 10.22 ml, very close to ovalbumin, which served as a standard protein (MW = 44,000 Da) (Figure 4C). The apparent MW of these proteins, calculated from the calibration curves generated for elution of molecular weight standards, correspond to molecular mass of 38,019 Da for dVHL and 40,833 Da for pVHL. These empirical values are larger than the calculated molecular mass of both proteins (19,000 Da) and are similar to the previously published values for pVHL monomers [34], [35]. The R_s_ values of dVHL and pVHL, calculated from the calibration curve are 2.64 nm and 2.93 nm, respectively (Figure 4D). Taken together, these results indicate that soluble pVHL and dVHL, unbound to their partners (Elongin B, C and HIF1-α) are larger than their calculated molecular weight (∼40 kDa vs. ∼19 kDa, respectively). These results together with our near CD spectra results suggest that both proteins have a molten globule conformation.

Affinity of proteins in a molten globule conformation to the hydrophobic probe ANS (8-anilino-1naphthalene sulfonic acid) is much stronger than in the rigid fully folded or the completely unfolded state [57], as reflected by a stronger fluorescence signal. We used ANS to assess the compactness and rigidity of packing of the hydrophobic core of dVHL. Figure 4E shows that at 25°C ANS fluorescence greatly increased upon binding of dVHL. The emission spectrum indicated that dVHL, as reported for pVHL [34], has a loosely packed hydrophobic core, and could comprise a molten globule tertiary structure. We monitored ANS fluorescence intensity at different temperatures (Figure 4E). The results indicate that at 4°C the accessibility of the hydrophobic core of dVHL to the solvent is slightly reduced due to the low temperature. At 37°C and 50°C (tertiary structure thermal denaturation) the protein is unfolded and loses the packing of the hydrophobic core. It is apparent that, as found in our CD spectra studies (Figure 4A) the transition between folded and unfolded conformation of dVHL resulting from thermal denaturation is irreversible, as previously reported for pVHL [34].

Collectively, the results from the CD spectra measurements, SEC data and ANS packing assessment indicate that dVHL and pVHL share similar folding, structure and stability. These *in vitro* findings mirror our *in silico* results which predict high similarity between the folding and structure of these proteins.

### Functional characterization of dVHL and pVHL

#### *In vitro*

To assess functional homology between dVHL and pVHL, we compared their ability to bind proline-hydroxylated ODD using an ELISA-based binding assay. For this purpose we synthesized two rhodamine-labeled peptides, termed Hyp402-ODD-HIF and Hyp850-ODD-SIMA, comprising the amino acid sequences surrounding P402 in HIF1-α and P850 in SIMA in which these proline residues were hydroxylated. Increasing concentrations of the two peptides were incubated, with either soluble dVHL, pVHL or with BSA as a negative control, and fluorescence intensity was measured.

The dVHL and pVHL proteins bound the Hyp402-ODD-HIF or Hyp850-ODD-SIMA peptides with similar affinities (Figure 4F). Both proteins showed higher affinity towards the HIF1-α-derived peptide than to the SIMA-derived peptide. pVHL bound both ODD peptides ∼1.5 fold stronger than dVHL.

#### *In vivo*

**Figure 5:**
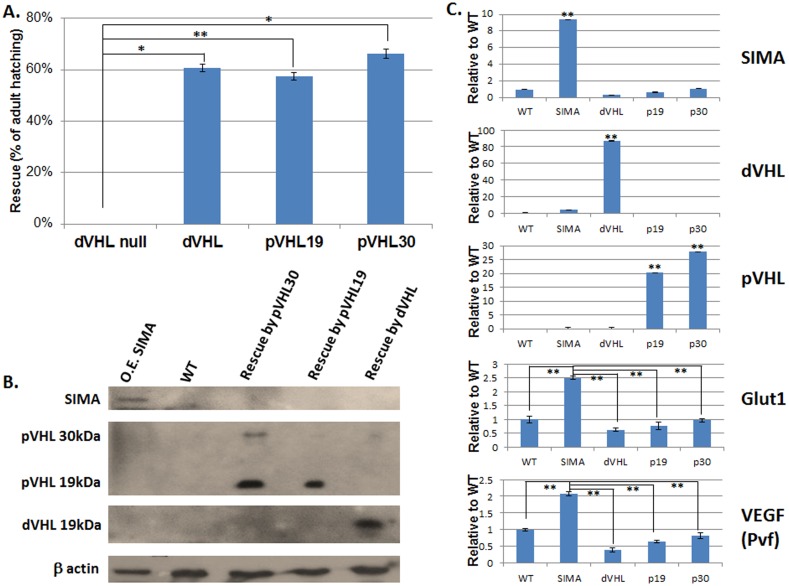
Rescue of the lethal *dVHL* null *Drosophila* by over-expression of either dVHL, pVHL19 or pVHL30. (**A**) **Percentage of surviving adult flies** (eclosing from the pupal case) relative to sibs that where heterozygous for the *dVHL* null mutation which served as a control. Rescue of homozygote *dVHL* null mutants by over-expression of either wild type dVHL or pVHL isoforms. Error bars  =  SEM of three independent experiments (n = 30 in each). (**B**) **Western blot detection** of SIMA, pVHL and dVHL in flies rescued by over-expression of either dVHL, pVHL19 or pVHL30. As controls we used wild type flies (WT) and flies over expressing (O.E.) the SIMA protein (180 kDa). We used anti SIMA antibody; *upper panel*, anti pVHL antibody; *second panel*, anti dVHL; *third panel* and anti β-actin; l*ower panel*. (C) Real-time qPCR analysis of *SIMA, dVHL, pVHL, Glut1* and *VEGF* (*pvf*) transcripts in the rescued flies. Expression of each gene was normalized by dividing the average gene value by the average value of the rp49 gene which was used as an internal control. The relative changes in mRNA expression of each gene were determined as x-fold changes relative to the wild type control (WT). Bars represent SEM (n = 5). P values were calculated by using the t test; *p<0.05; **p<0.01.

In order to assess if this evolutionarily conserved binding of the target ODD takes place also in the intact organism. We next examined whether pVHL can rescue the mutant phenotype of *Drosophila* lacking endogenous dVHL. Larvae homozygous for a null mutation in the *Drosophila* VHL exhibit characteristic slugginess evident immediately after hatching and die at the end of first instar larval stage [70]. Ubiquitous expression of either dVHL or pVHL transgenes (encoding the 19 kDa or the 30 kDa isoforms of pVHL) by tubulin Gal4 lead to ∼60% of the animals completing development and eclosing as adult flies, indicating functional complementation (Figure 5A). To further examine whether this rescue reflects restoration of the HIF1-α pathway in these flies, we examined the level of the endogenous fly HIF1-α homolog protein, SIMA. Rescue of the null *dVHL* phenotype would suggest that the transgene is capable of normal targeting the fly HIF1-α homolog (SIMA) for degradation (as in wild type flies). Total protein extraction was prepared from the rescued and wild type flies, as well as from flies over expressing SIMA, and were immunoblotted with anti-SIMA antibodies. The SIMA protein level in the flies rescued by dVHL or by either isoform of pVHL, was found to be similar to its level in wild type flies and considerably lower than in flies over-expressing SIMA which served as a control (Figure 5B). This is in agreement with our *in vitro* study which indicated that the two proteins directly bind the ODD of the SIMA protein with similar affinities. In addition, we examined in these flies the level of expression of the SIMA target genes Glut1 and VEGF [20], [58], [59]. Real time qPCR indicated that the level of the transcripts of these genes in flies rescued by either dVHL or by the isoforms of pVHL is similar to their level in wild type flies (Figure 5C). These results indicate that pVHL is able to directly bind SIMA in the fly milieu and to properly regulate its downstream pathways.

**Figure 6:**
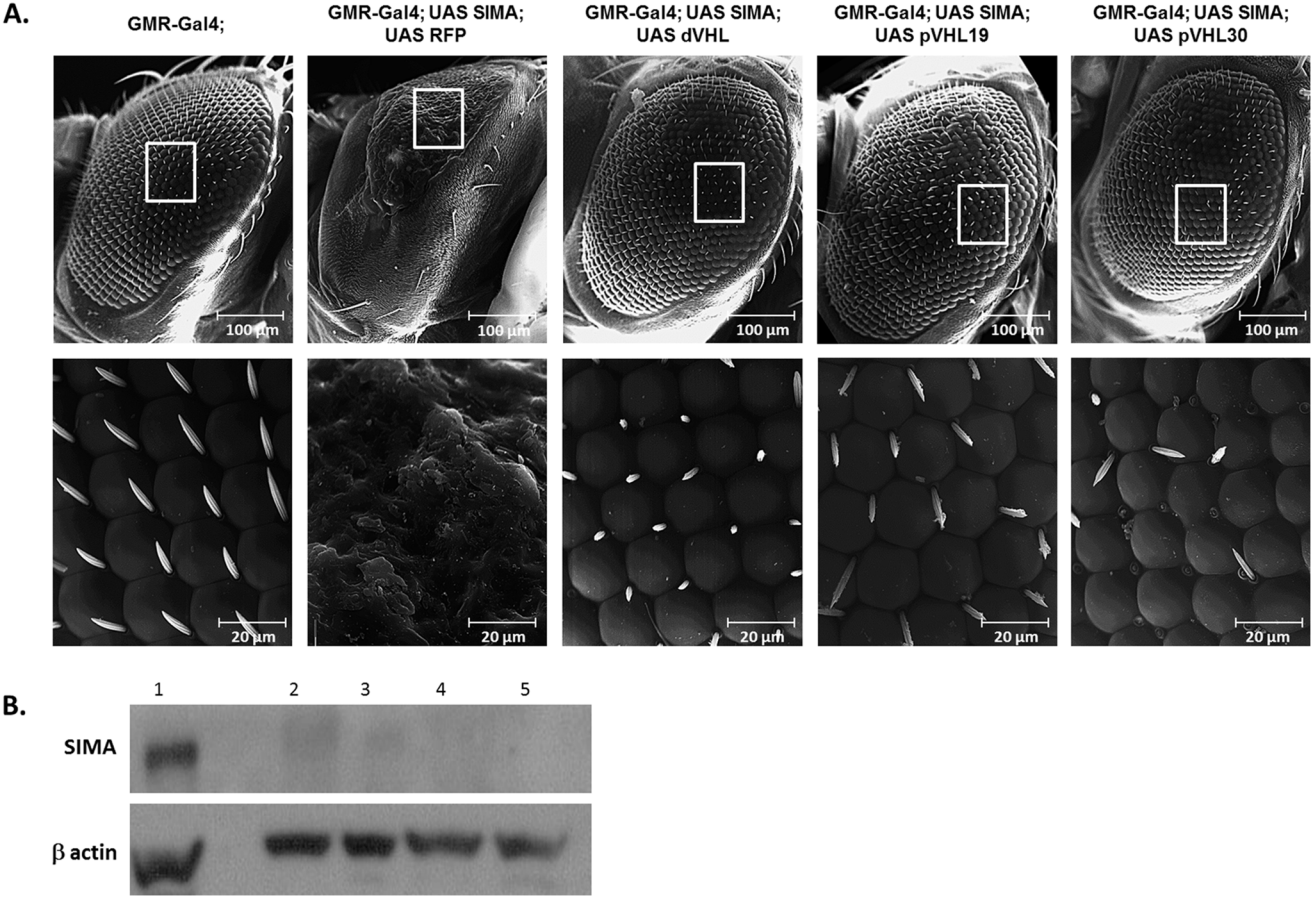
The eye defect caused by over-expression of SIMA is suppressed by co-expression of either dVHL, pVHL19, or pVHL30. (**A**) **SEM images** of *Drosophila* eyes at x500 magnification. Genotypes: GMR-Gal4 (left), GMR-Gal4; UAS-SIMA; UAS-RFP (second from left), GMR-Gal4; UAS-SIMA; UAS-dVHL (third from left) GMR-Gal4; UAS-SIMA; UAS-pVHL19 (fourth from the left) and GMR-Gal4; UAS-SIMA; UAS-pVHL30 (right). Scale bar: 100 µm upper panel and 20 µm lower panel. (**B**) **Western blot** **analysis** showing the level of SIMA in proteins extracted from flies heads. β-actin was used as loading control. (**1**) GMR-Gal4; UAS-SIMA; UAS-RFP. (**2**) GMR-Gal4. (**3**) GMR-Gal4; UAS-SIMA; UAS-dVHL. (**4**) GMR-Gal4; UAS-SIMA; UAS-pVHL19. (**5**) GMR-Gal4; UAS-SIMA; UAS-pVHL30.

To further demonstrate that the human protein, pVHL is capable of normal regulation of the fly HIF1-α homolog SIMA we over-expressed SIMA in the *Drosophila* eyes using the GMR-Gal4. Over-expression of SIMA resulted in a rough-eye phenotype in adult flies, evident by loss of regularity of the lattice-like organization of the ommatidia (Figure 6A). When SIMA was co-expressed in the eyes together with dVHL, as a control, or with either pVHL19 or pVHL30 the rough-eye phenotype was rescued (Figure 6A). At 29°C over-expressing SIMA in the eye resulted in lethality at the pupal stage, while when co-expression with either dVHL of pVHL the lethality was rescued (movie S1). This rescue was accompanied by reduction in the level of SIMA to the level typical of normal flies (Figure 6B). These results indicate that pVHL recognizes SIMA in the fly and targets it for degradation as does dVHL.

**Figure 7:**
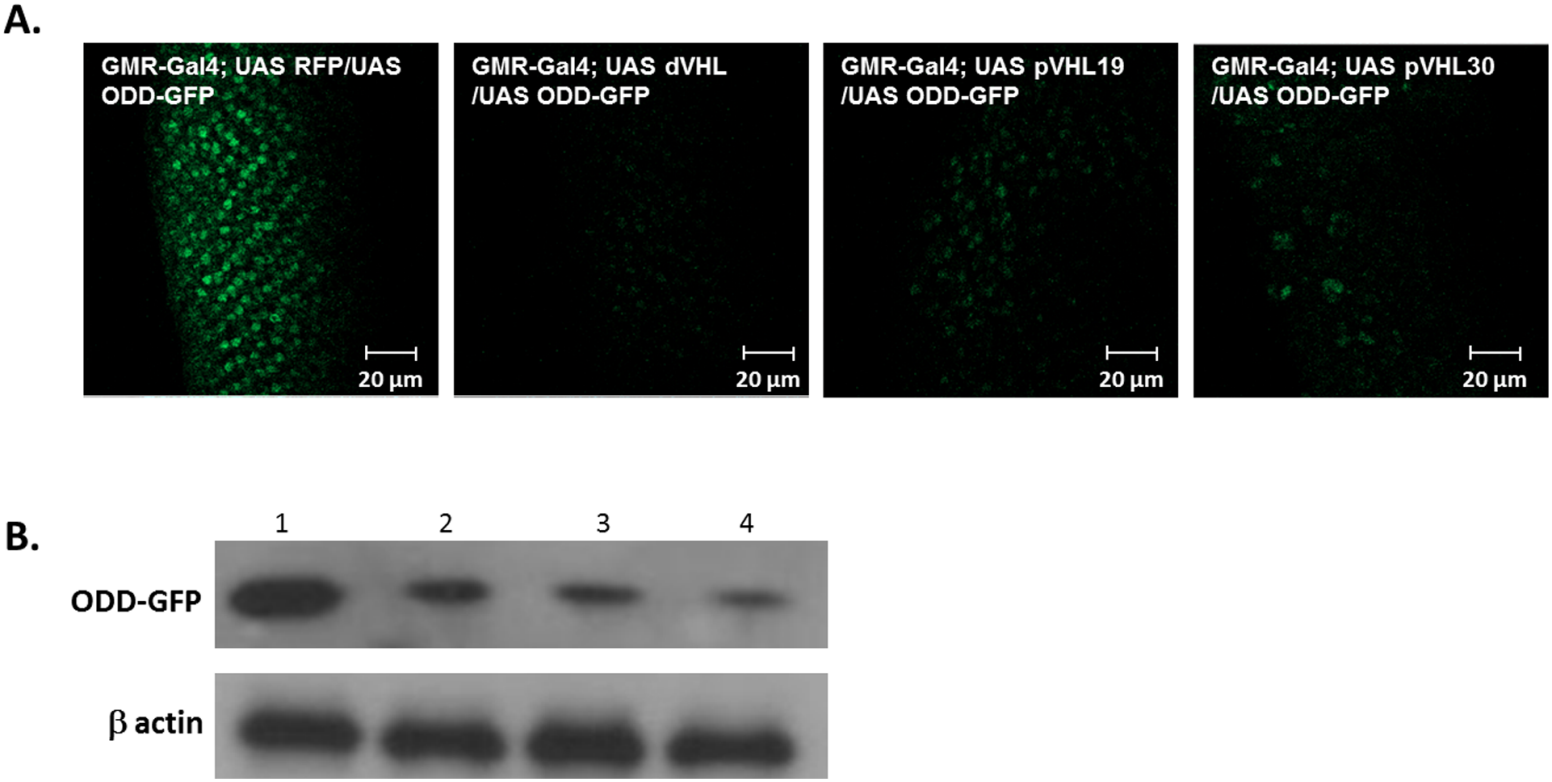
The GFP fluorescence signal caused by over-expression of ODD-GFP is lowered by co-expression of either dVHL, pVHL19, or pVHL30. (**A**) **GFP levels** were analyzed by confocal microscopy. Genotypes: GMR-Gal4; UAS-ODD-GFP/UAS-RFP (left), GMR-Gal4; UAS-ODD-GFP/UAS-dVHL (second from left) GMR-Gal4; UAS-ODD-GFP/UAS-pVHL19 (third from the left) and GMR-Gal4; UAS-ODD-GFP; UAS-pVHL30 (right). Scale bar, 20 µm (**B) Western blot** analysis showing the levels of ODD-GFP protein extract from flies heads. β-actin was used as loading control. (**1**) GMR-Gal4; UAS-ODD-GFP/UAS-RFP. (**2**) GMR-Gal4. (**3**) GMR-Gal4; UAS-ODD-GFP/UAS-dVHL. (**4**) GMR-Gal4; UAS-ODD-GFP/UAS-pVHL19. (**5**) GMR-Gal4; UAS-ODD-GFP/UAS-pVHL30.

In a reciprocal experiment, we tested whether dVHL or pVHL can recognize, in the fly, ODD - the hallmark fragment of HIF1-α (see Introduction). When co-expressed in the fly eye imaginal discs together with either isoform of pVHL, the GFP signal of the ODD-GFP fusion protein was markedly reduced in comparison to control flies (Figure 7A). This reduction was similar to that caused by co-expression of ODD-GFP and dVHL (Figure 7A, B), as previously reported [43]. Western blot analysis of extracts from heads of the corresponding adults verified that the reduced GFP signal reflected lower levels of the ODD-GFP protein in the presence of dVHL or either isoform of pVHL (Figure 7B). These results indicate that under normoxic conditions both dVHL and pVHL recognize the ODD domain of HIF1-α and target it for degradation.

**Figure 8:**
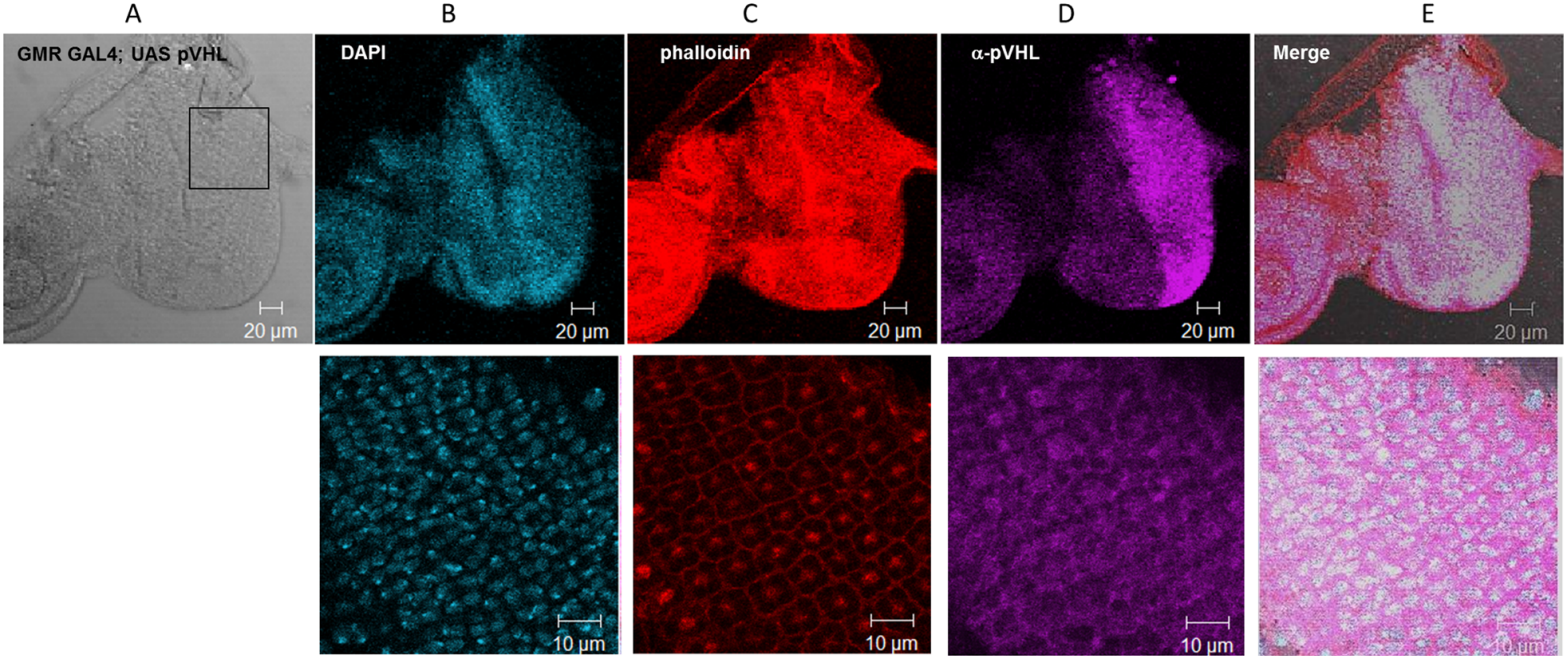
Subcellular localization of pVHL in transgenic Drosophila. Immunofluorescence staining of the *Drosophila* eye imaginal discs over expressing pVHL30 by GMR Gal4. (**A**) Bright field (**B**) Nuclei stained using DAPI (blue) (**C**) Actin stained using phalloidin 568 (red) (**D**) Anti-pVHL antibody was used for identification of pVHL protein (purple). (**E**) Merge. Upper panel scale bar: 20 µm; lower panel scale bar: 10 µm (zoom in).

To further verify that pVHL functions normally in the fly milieu, we monitored its subcellular localization. In cells of the eye imaginal discs the transgenic pVHL was found both in the cytoplasm and in the nucleus as reported for this protein in mammalian cells [60] (Figure 8).

Overall our results demonstrate structural and conformational similarity between dVHL and pVHL underlining their functional homology. Not only are they able to directly bind the reciprocal binding partners, but pVHL is able to replace dVHL in *Drosophila*, while keeping the HIF1-α pathway intact.

## Discussion

Lower model organisms such as *Drosophila melanogaster* and *Caenorhabditis elegans* have often provided the first glimpse into the mechanism of action of human cancer-related proteins, thus making a substantial contribution to the elucidation of the molecular basis of the disease. More than 50% of the proteins that are associated with human diseases, including cancer, have orthologues in *D. melanogaster* [61]–[63]. In many cases, the function of a given protein is also conserved evidenced by the fact that the corresponding human protein can rescue the loss of function of its *Drosophila* orthologue [64]. Thus, studies of the fly orthologue as well as investigation of the transgenic human proteins in flies, which are highly amenable for genetic manipulations, provide important information on the human disease-associated protein and the pathways it is involved in. This is exemplified especially regarding to the contribution of *Drosophila* to basic and applied aspects of cancer research (for recent reviews see [65], [66]). The highly ordered, lattice-like, arrangement of the ommatidia in the fly eyes is especially suitable for large scale screens for genetic modifiers and for drug candidates [64]–[66]. Flies have also greatly facilitated drug screening [64]. Such studies have shown that the *Drosophila* VHL (dVHL) can interact with *Drosophila* Elongin C *in vitro* [40], [41] and form a complex with the human Elongin CB complex and mouse Rbx-1, but not with the human Elongin A. Thus, the ubiquitin ligase function of pVHL is conserved in *Drosophila*. Subsequently, Irisarri et al. (2009) and Arquier et al. (2006) demonstrated *in vivo* that dVHL is involved in degradation of hydroxylated oxygen-dependent degradation domain (ODD) of both the human HIF1-α or of the *Drosophila* homolog, SIMA. Studies of dVHL have suggested the involvement of the VHL protein in regulation of endocytosis and epithelial cell motility which was subsequently demonstrated for pVHL as well [67]–[69]. Research on dVHL has also indicated a role for the VHL protein in microtubule stabilization during epithelial morphogenesis [70]. Yet, this remains to be demonstrated for pVHL as well.

The results presented here regarding the structural similarity between the dVHL and pVHL proteins provide insights into their functional homology. We have shown that pVHL is a molten globule protein [34], and have subsequently demonstrated that it is an intrinsically disordered protein (IDP) and is functional as such [35]. Here we show that dVHL, like pVHL, has a secondary structure but lacks well-defined tertiary structure and attains a molten globule conformation. Our results indicate that dVHL, like pVHL, is functional without being uniquely folded into a rigid 3D structure. Thus dVHL like pVHL is an IDP.

The demonstration of homology between IDPs is a major endeavor since they do not display sequence conservation. In addition, due to the lack of well-defined 3D structure, IDP regions fail to scatter X-rays coherently, hampering attempts to solve their structure by X-ray crystallography [71]–[73].

The *in silico* predicted homology in the conformation of the key functional amino acids of dVHL and pVHL, as well as their evolutionary conservation, supported by our *in vitro* results, provide a structural basis for understanding the homology in their function as well as their ability to interact with each other’s binding partners. dVHL can bind *in vivo* the hydroxylated ODD domain of the human HIF1-α and target it for degradation [43] and here we showed that transgenic pVHL can do the same in flies. Moreover, we demonstrated that transgenic pVHL can rescue the lethal phenotype of dVHL null mutants of *Drosophila.* These results suggest that pVHL is capable of interacting, in flies, with the *Drosophila* members of the VCB complex, thus regulating the fly HIF1-α homolog, SIMA. Indeed, we have shown that this rescue flies involves regulation of the HIF1-α/SIMA dependent pathway, evidenced by the level of its target genes.

The *Drosophila* model described in the present work should facilitate further studies on the function of pVHL and the pathways it regulates. The rough eye phenotype caused by over-expression of SIMA, which we have shown to be suppressed by transgenic expression of normal pVHL, should be highly conducive for conducting screens for drug candidates for the VHL syndrome in Drosophila whole model organism. These, can be subsequently verified molecularly by examining the downstream HIF1-α dependent pathway which are conserved between flies and humans.

## Materials and Methods

### *In Silico* Analysis

The amino acid sequences of human and *Drosophila melanogaster* VHL proteins were obtained from Uniprot [74]. Pairwise alignment of these two sequences was constructed using the MUSCLE algorithm [47]. As these two orthologs show only <22% sequence identity and <30% similarity, we utilized fold recognition and homology detection servers in order to find structural templates for *Drosophila* VHL. The FFAS03 fold recognition server identified the structure of human pVHL19 (PDB ID 1LM8) as the best template with a significance score of –77.3 [45]. The HHpred remote protein homology detection and structure prediction server also identified human pVHL19 as a suitable template [46]. Sequence alignments were constructed using the MUSCLE algorithm and manually corrected based on data from FFAS03 and HHpred as well as PSIPRED (for secondary structure predictions) and ConSurf (for evolutionary conservation considerations) [49]–[52], [75]. The models were constructed by MODELLER with the best one selected based on structural data regarding key structural and functional amino acids as well as hydrophobicity [53]. Additionally, the quality parameters of each model were estimated with ProSA and Procheck [76], [77]. The coordinate file (PDB format) of our model structure can be found at doi:10.5061/dryad.14pp7.

### Gene Subcloning

The open reading frame of pVHL, kindly provided by Dr. Nikola Pavletich (Memorial Sloan-Kettering Cancer Center, New York), and the dVHL ORF following reengineering to adapt it to the *E. coli* codon usage for obtaining better yield, were cloned into the pET14b vector (Novagen) using *Nde*I and *Xho*I (TaKaRa) restriction enzymes.

### Proteins Expression and Purification

The vectors were transformed to Rosetta strain of *E. coli*. Transformed cells were grown at 37°C, 200 rpm, in 2XYT medium (Difco) under antibiotic selection (100 µg/ml ampicillin). The cells were grown to an OD_600_ = 2.5 and induced by adding 1 mM of Isopropyl β-D-1-thiogalactopyranoside (Sigma-Aldrich) for 3 hours at 37°C. The pVHL and dVHL proteins were found to be insoluble in the bacterial inclusion bodies. The cells were broken using a high-pressure homogenizer and adding 40 mg of lysozyme (Sigma-Aldrich) in TE 50∶20 buffer (50 mM Tris-HCl pH 8, 20 mM EDTA). Following disruption, the inclusion bodies were recovered and washed in TE 50∶20 buffer with 1%v/v Triton using centrifugation. Inclusion bodies pellet was dissolved in the presence of 6 M guanidinium hydrochloride and diluted to 10 mg/ml protein concentration. The protein was refolded from fully reduced and unfolded samples by dialysis in step-wise decreasing concentrations of the denaturant guanidine hydrochloride to working buffer (10 mM Tris-HCl pH 8.5, 200 mM NaCl). pVHL and dVHL were purified to >97% purity, and migrated on SDS-PAGE as a 19 kDa proteins. Protein concentration was determined from absorbance at 280 nm using ε280 nm = 18450 cm^−1 ^M^−1^ for pVHL and ε280 nm = 33920 cm^−1 ^M^−1^ for dVHL.

### Size Exclusion Chromatography (SEC)

Gel filtration experiments were performed using Superdex75 HR 10/30 size exclusion column (GE Healthcare) with a separation range of 3–70 kDa connected to an FPLC prime automated liquid chromatography system (Amersham Biosciences). The running buffer used was 10 mM Tris-HCl pH 8, 200 mM NaCl. The column was calibrated using gel filtration molecular weight standard (Bio-Rad). The following standards were used for calibration: Thyroglobulin (670 kDa, Rs = 8.6 nm), γ-globulin (158 kDa, Rs = 5.1 nm), Ovalbumin (44 kDa, Rs = 2.8 nm), Myoglobin (17 kDa, Rs = 1.9 nm), Vitamin B12 (1.35 kDa), and Dextran blue (2 MDa).

A 0.5 ml protein sample at a final concentration of 5 µM was filtered and chromatographically analyzed using a flow rate of 0.5 ml/min. Absorbance was monitored at 280 nm, elution volumes were determined from UV chromatogram. The partition coefficient, Kav, was calculated from the elution volume of the sample, Ve, and total bed volume, Vt, using the expression: 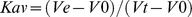. Stokes radius (Rs) for all proteins was calculated by plotting 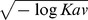 versus known Rs. Calibration curves and equations were established.

### CD Spectra Measurements

CD spectra at far- (200–250 nm) and near-UV (250–320 nm) were recorded with an Chirascan CD Spectrometer (Applied Photophysics) equipped with a temperature-controlled cell using a cell of path length 0.5 cm; bandwidth was 1 nm, and averaging time was 30 s for each measurement. Protein concentration was 5 µm in a buffer containing 10 mM Tris-HCl (pH 8) and 200 mM NaCl.

### 8-Anilino-1-naphthalene Sulfonic Acid (ANS) Fluorescence Studies

Fluorescence emission spectra of two solutions were generated with excitation wavelength of 350 nm. The first solution contained 5 µM ANS in buffer (10 mM Tris-HCl, pH 8, and 200 mM NaCl). The second solution was similar and contained protein at a final concentration of 5 µM. Samples were equilibrated at room temperature. The fluorescence emission spectra were recorded (Horiba Jobin Yvon FL3-11 Spectrofluorometer). The cuvette length was 1 cm. Measurements were conducted at 4, 25, 37, and 50°C. Baseline corrections were made with buffer lacking protein and ANS.

### Protein function

An ELISA-based binding assay was used. The peptide substrates were: SIMA (SFEAFAGRAPYIPIDDD), HIF1-α P402 (DALTLLAPAAGDTIISLDF). These target peptides were synthesized by Hy Laboratory Ltd (Israel). They correspond to residues 841–857 of SIMA, and residues 395–413 of HIF1-α, respectively. These peptides comprised the amino acid Proline and had this residue hydroxylated to be recognized by the VHL proteins. The peptides were tagged with the fluorophore tetramethylrhodamine (TAMRA). pVHL or dVHL were diluted in salt buffer (10 mM Tris-HCl, pH 8, 200 mM NaCl and 200 mM GnHCl) to 10 µg/ml. 100 µl of the tested protein were add to each well of an ELISA plate (Costar EIA). Experiments were performed in triplicates and error bars are presented. Plates were incubated overnight at 4°C. Wells were washed four times with high salt buffer. Blocking buffer (high salt buffer containing 5% w/v BSA) was added to all wells to block protein binding sites left open in the wells. Labeled Hyp402-ODD-HIF or Hyp850-ODD-SIMA target peptide solutions, at increasing concentrations, were added to the wells. Plates were incubated for 1 h at 25°C and were washed thereafter four times with buffer. Fluorescence intensity at λ_580 nm_ was measured following excitation at λ_540 nm_. BSA protein (Amresco) served as a negative control.

### Flies and crosses

Wild-type (wt) (Oregon R) and mutant strains were reared on standard cornmeal-molasses medium at 25°C. Experiments were carried out at 25°C or 29°C as indicated. Adult offspring (F1) from the crosses were collected up to 9 days after the beginning of their eclosion at 25°C in order to avoid offspring from the next generation (F2).

### *Drosophila* strains and genetics

Oregon-R was used as the wild-type stock in this study. The *Drosophila dVHL* ^1^ mutant was generated by replacing the wild-type copy with a deletion that removes 81 codons encompassing the first two in-frame AUGs (a generous gift from T. Hsu, Boston University, USA). The cDNA of *dVHL*, *pVHL19* and the full length *pVHL30* variant (carrying the extra 51 amino acids at the N terminal of the protein) were cloned in the UAS-based pUAST vector using *Eco*RI and *Xho*I restriction enzymes. These expression vectors were used to transform the *w^1118^* flies (BestGene, CA). We used transgenic strains carrying stable insertions on chromosome 3 that showed the highest protein expression. Tubulin-Gal4 was kindly provided by O. Gerlitz (Hebrew University of Jerusalem, Israel), and used for inducing UAS-dependent ubiquitous expression. GMR-Gal4 was used to drive expression of VHL in the eye imaginal disc cells. UAS-ODD-GFP transgenic strains carrying stable insertion on chromosome 3 were used [43]. Unless specified otherwise, all fly stocks are from the Bloomington Stock Center.

### Antibodies

Rabbit anti-dVHL antibodies were obtained from Adar Biotech (Israel), using dVHL immunogenic selected peptide (IRVRVPKSQQSPDQL). The polyclonal antibodies were characterized using a western blot analysis. Rabbit polyclonal antibodies were obtained from the following sources: anti-GFP antibody (Abcam), anti-pVHL (Abcam) and anti-SIMA antibody (generous gift from P. Wappner, Instituto Leloir, Buenos Aires, Argentina). Mouse monoclonal anti-β-actin (Abcam). Secondary antibodies used were from Santa Cruz Biotechnology (horseradish peroxidase-coupled goat anti-mouse and goat anti-rabbit antibodies). For immunofluorescence experiments, pVHL was detected with a CY5 conjugate anti-rabbit antibody (Santa Cruz Biotechnology).

### Protein extraction

Collected flies or fly heads were homogenized in 1∶1 volume of either extraction buffer (5 mM MgCl_2_, 5 mM DTT, 1 mM PMSF, 1×Complete protease inhibitor cocktail (Roche), 1×PBS, pH 7.5). The samples were centrifuged for 30 min at 14,000 rpm at 4°C. Protein concentration was measured using Bradford reagent (Sigma). Laemmli sample buffer (BioRad) was added to 40–100 µg of protein from each sample, followed by boiling for 10 min, centrifugation for 5 min at 14,000 rpm and analysis by SDS-PAGE and Western blot.

### Immunoblotting

Protein extracts were separated by 4–20% gradient SDS-PAGE (GeBA, Israel) and transferred on to PVDF using iBlot 7-Minute Blotting System (Life Technologies). The membrane was blocked for 1 h in blocking solution (5% milk powder, 0.02% sodium-azide in 1×TBS), and then incubated in the primary antibody diluted in blocking solution. The membrane was then washed 3 times for 15 min each in TTBS (0.1% Tween-20 in 1×TBS), incubated for 40 min in the secondary antibody and washed 3 times for 10 min in TTBS. The membrane was developed using EZ-ECL (Biological Industries), according to the manufacturer’s instructions, and exposed to Fuji Medical X-Ray Film for up to 5 min. Films were developed using Kodak X-OMAT 2000.

### Immunofluorescence staining

Females or males larvae at the 3^rd^ instar wandering stage were dissected in PBS for separation of the eyes imaginal discs. The separated tissue was fixed for 20 min in fixation solution (4% paraformaldehyde in PBS) and washed 3 times in PBT (0.3% Triton in PBS). The tissues were then blocked for 1 h in blocking solution (5% BSA in PBT) and incubated over night at 4°C in 200 µl of the primary antibody diluted 1∶50 in blocking solution and containing 1∶100 red phalloidin (Life Technologies). The samples were washed 3 times for 10 min each in PBT, incubated for 2 h in the secondary antibody 1∶200 and washed 3 times for 10 min in PBT. After being thoroughly washed with PBS, cells were mounted using ProLong Antifade containing DAPI (Invitrogen). Images were taken with LSM510 confocal microscope (Zeiss).

### Reverse Transcription and Real-time PCR

Total RNA from 10 adult flies was isolated with RNeasy mini kit with on-column DNase digestion (Qiagen). First-strand cDNA was generated by using the Verso cDNA Kit (Thermo). Real-time PCR was performed in triplicate with KAPA Fast SYBR master mix (KAPA Biosystems) (Qiagen) and the StepOnePlus Real-Time PCR Systems (Life Technologies). Primers for rp49, dVHL, pVHL, SIMA, Glut1 and VEGF (pvf in flies) were purchased from Hy labs (Israel). All values were normalized to the level of rp49 mRNA abundance and to the wild type (Oregon R) flies. Each primers pair was calibrated using the Absolute Quantification program with increasing concentrations of cDNA, from 1∶32 to 1∶1 dilutions of the original cDNA. Primers used: rp49 (forward 5′- ACC GAT GTT GGG CAT CAG ATA-3′; revers 5′- TAA GCT GTC GCA CAA ATG GC-3′). SIMA (forward 5′- CTG CCC GAG AGC AAT CCG TA-3′; revers 5′- CGG TGG TGT TAG GGG TGG AG-3′). dVHL (forward 5′- CAG AGT CCG GAT CAG CTG GTT GAC G-3′; revers 5′- GAC TCG ATG CAG GTC AGC AGG CTG-3′). pVHL (forward 5′ CCT CCC AGG TCA TCT TCT GCA AT-3′; revers 5′- GTT AAC CAG AAG CCC ATC GTG TG-3′). Glut1 (forward 5′- ACC TAC TCG ATT TTC TCG GCG GTG-3′; revers 5′- GCC CAG CAT GCC GCC GAT-3′). VEGF pvf (forward 5′- ATG CAG CAT CCA TTG CTC ATC CTG C-3′; revers 5′- TCC TTG GCA GCA GCT GCC CTC-3′).

### Scanning electron microscopy (SEM)

SEM was performed as described [78]. All SEM images from at least three female adult eyes of each genotype were analyzed.

## Supporting Information

Figure S1 **Superposition of the novel model structure produced by our composite approach with models produced by standard modeling tools.** The model structure produced by our composite approach (blue) shares significant structural similarity to the models produced by I-TASSER (yellow) and Phyre2 (green).
(TIF)

Figure S2 **Superposition of the amino acids involved in dVHL interactions with its binding partners.** **(A)** Superposition of the key amino acids involved in HIF1-α binding. These are residues W70, H68, T64, and Y51and W41 in dVHL. **(B)** Superposition of the key amino acids involved in Elongin C binding. These are residues L154, L134, V130, C126 and L122 in dVHL. **(C)** Superposition of the key amino acids involved in Cul2 binding. These are residues L158, L154 and I150 in dVHL. The orientation of these key amino acids as predicted by our composite approach (blue) is similar to that of the corresponding amino acids of the models produced by I-TASSER (yellow) and Phyre2 (green).
(TIF)

Figure S3 **Pairwise sequence alignment of the** ***Drosophila*** **and Human Elongin C orthologs.** Pairwise sequence alignment between human Elongin C and Drosophila Elongin C, as calculated by the MUSCLE algorithm, showing conservation of the hydrophobicity between the two proteins. Hydrophobic residues (red), neutral residues (purple) and hydrophilic residues (blue).
(TIF)

Figure S4 **Thermal denaturation assay.** Thermal denaturation of wild type dVHL. **(A)** Denaturation. Changes in ellipticity monitored at 200–250 nm. Dashed line marks the Tm of dVHL. **(B)** Renaturation. Changes in ellipticity monitored at 200–250 nm.
(TIF)

Movie S1 **The eye defect caused by over-expression of SIMA is suppressed by co-expression of either dVHL, pVHL19, or pVHL30 at 29°C.** **1.** GMR-Gal4; UAS-SIMA; UAS-RFP (no hatching). **2.** GMR-Gal4; UAS-SIMA; UAS-dVHL. **3.** GMR-Gal4; UAS-SIMA; UAS-pVHL19. **4.** GMR-Gal4; UAS-SIMA; UAS-pVHL30. At 29°C over expression of SIMA by GMR-Gal 4 caused pupal lethality (1) that could be rescued by co-expression of either dVHL, pVHL19 or pVHL30 (evidenced as flies motility 2–4).
(WMV)
