# Supplementary figures and images for: Computational and Experimental Characterization of dVHL Establish a Drosophila Model of VHL Syndrome
Source: PLoS One. 2014 Oct 13;9(10):e109864. doi: 10.1371/journal.pone.0109864 (PMC4195687; doi:10.1371/journal.pone.0109864)

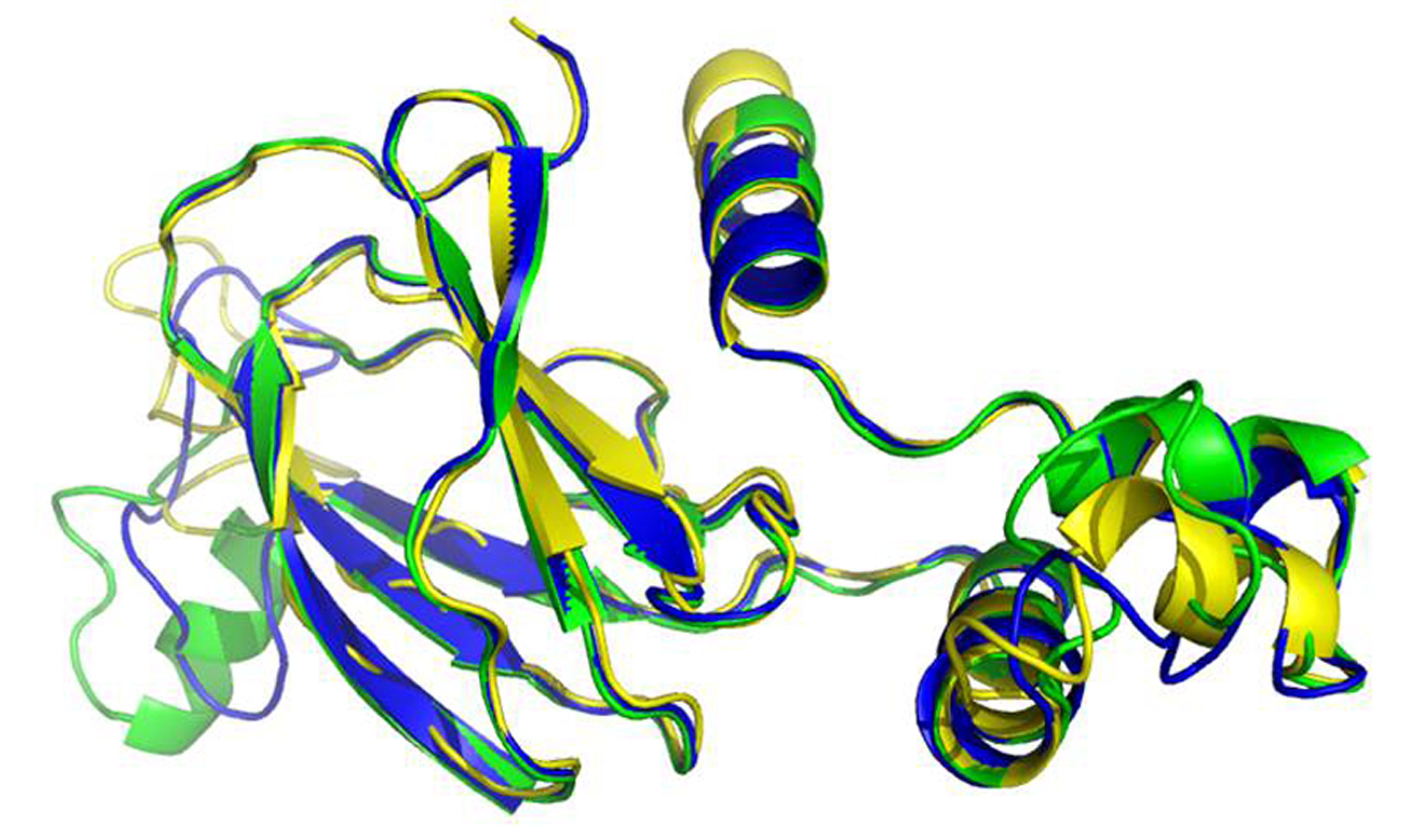

Supplement: Figure S1 — Superposition of the novel model structure produced by our composite approach with models produced by standard modeling tools. The model structure produced by our composite approach (blue) shares significant structural similarity to the models produced by I-TASSER (yellow) and Phyre2 (green). (TIF) [file pone.0109864.s001.tif]

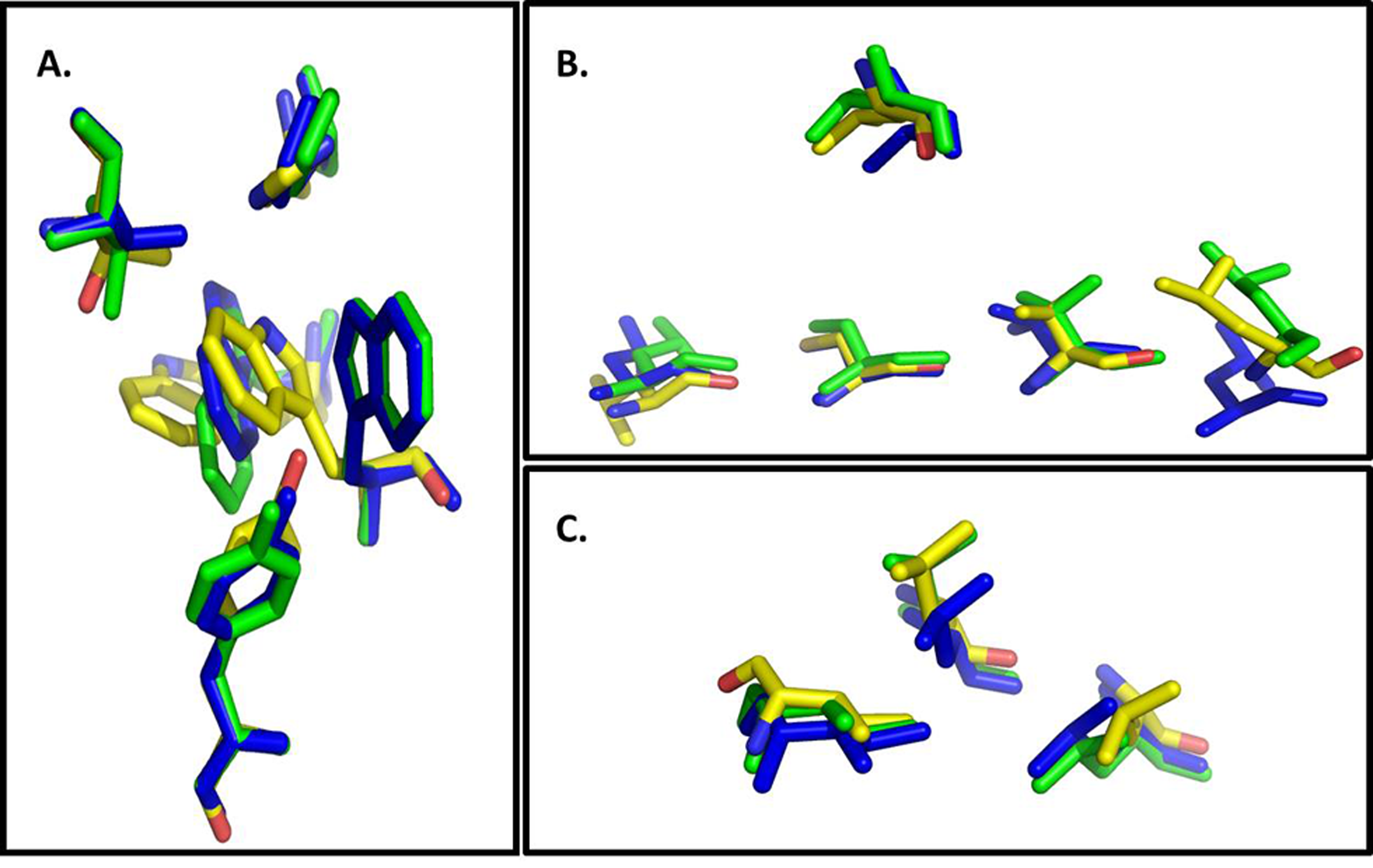

Supplement: Figure S2 — Superposition of the amino acids involved in dVHL interactions with its binding partners. (A) Superposition of the key amino acids involved in HIF1-α binding. These are residues W70, H68, T64, and Y51and W41 in dVHL. (B) Superposition of the key amino acids involved in Elongin C binding. These are residues L154, L134, V130, C126 and L122 in dVHL. (C) Superposition of the key amino acids involved in Cul2 binding. These are residues L158, L154 and I150 in dVHL. The orientation of these key amino acids as predicted by our composite approach (blue) is similar to that of the corresponding amino acids of the models produced by I-TASSER (yellow) and Phyre2 (green). (TIF) [file pone.0109864.s002.tif]

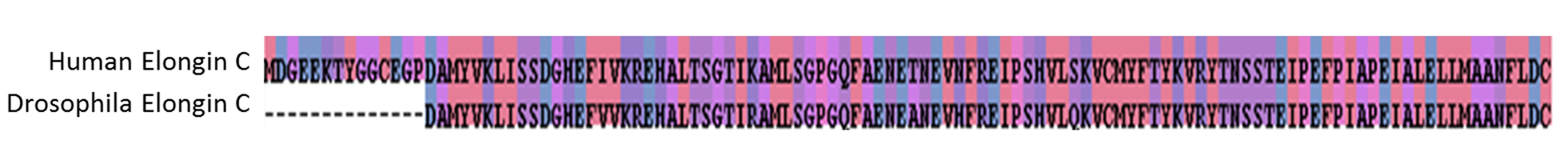

Supplement: Figure S3 — Pairwise sequence alignment of the Drosophila and Human Elongin C orthologs. Pairwise sequence alignment between human Elongin C and Drosophila Elongin C, as calculated by the MUSCLE algorithm, showing conservation of the hydrophobicity between the two proteins. Hydrophobic residues (red), neutral residues (purple) and hydrophilic residues (blue). (TIF) [file pone.0109864.s003.tif]

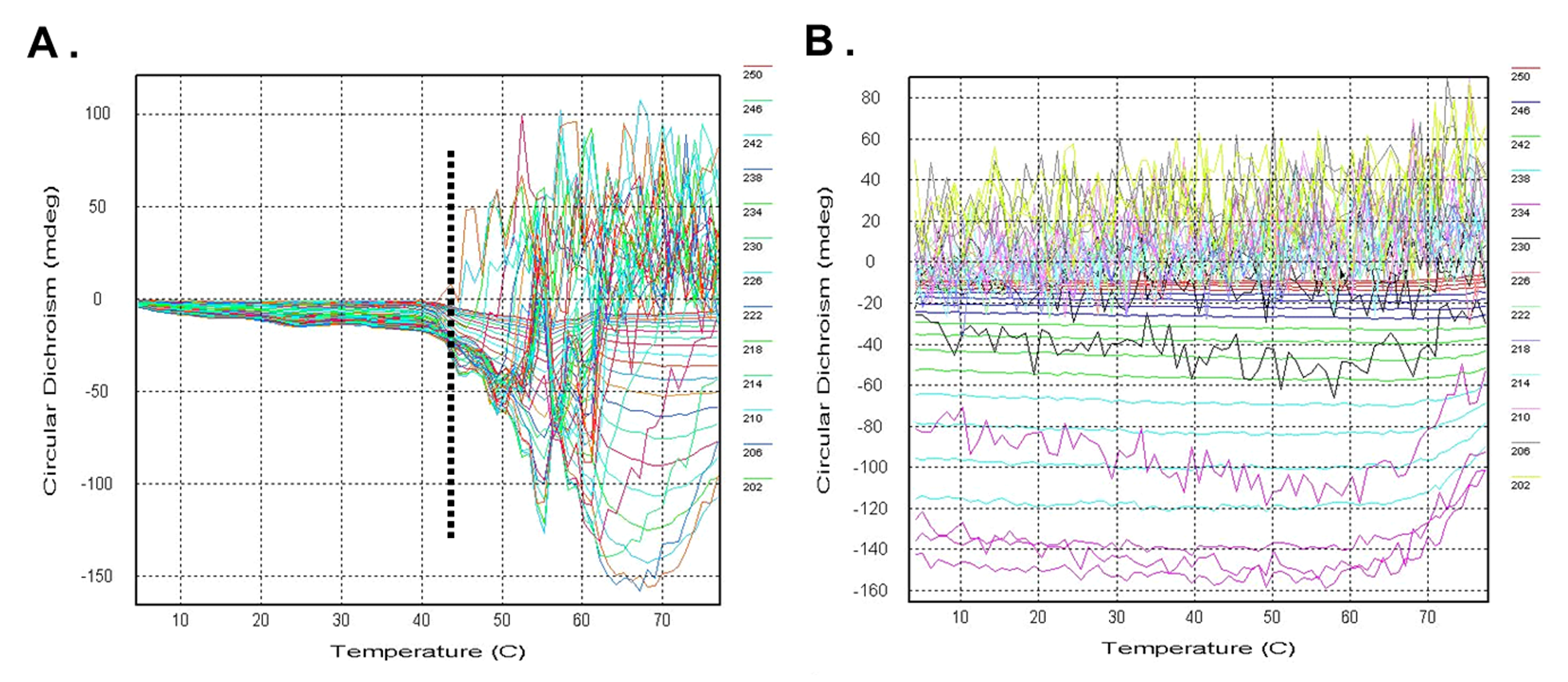

Supplement: Figure S4 — Thermal denaturation assay. Thermal denaturation of wild type dVHL. (A) Denaturation. Changes in ellipticity monitored at 200–250 nm. Dashed line marks the Tm of dVHL. (B) Renaturation. Changes in ellipticity monitored at 200–250 nm. (TIF) [file pone.0109864.s004.tif]
